# Hsa_circ_0040809 and hsa_circ_0000467 promote colorectal cancer cells progression and construction of a circRNA-miRNA-mRNA network

**DOI:** 10.3389/fgene.2022.993727

**Published:** 2022-10-20

**Authors:** Jingfu Liu, Shan Chen, Zhen Li, Wenhao Teng, Xianren Ye

**Affiliations:** ^1^ Department of Blood Transfusion, Clinical Oncology School of Fujian Medical University, Fujian Cancer Hospital, Fuzhou, China; ^2^ Department of Gastrointestinal Surgery, Clinical Oncology School of Fujian Medical University, Fujian Cancer Hospital, Fuzhou, China; ^3^ Fujian Provincial Key Laboratory of Tumor Biotherapy, Fuzhou, China

**Keywords:** circRNA, hsa_circ_0000467, hsa_circ_0040809, colorectal cancer, competing endogenous RNA

## Abstract

**Objective:** Circular RNAs (circRNAs) have been demonstrated to be closely involved in colorectal cancer (CRC) pathogenesis and metastasis. More potential biomarkers are needed to be searched for colorectal cancer (CRC) diagnosis and treatment. The objective of this study is to seek differentially expressed circRNAs (DEcircRNAs), test their roles in CRC and construct a potential competing endogenous RNA (ceRNA) network.

**Methods:** CircRNA microarrays were obtained from Gene Expression Omnibus, and differential expression was analyzed by R software. The relative expressions of DEcircRNAs were confirmed in CRC tissues and cell lines by qRT-PCR. MTs and Transwell experiments were performed for detecting the roles of circRNAs on CRC cell proliferation and migration, respectively. Targeted miRNAs of circRNAs and targeted mRNAs of miRNAs were predicted and screened by bioinformatics methods. A ceRNA network of DEcircRNAs was constructed by Cytoscape. To further verify the potential ceRNA network, the expressions of miRNAs and mRNAs in knockdown of DEcircRNAs CRC cells were detected by qRT-PCR.

**Results:** Two DEcircRNAs (hsa_circ_0040809 and hsa_circ_0000467) were identified and validated in CRC tissues and cell lines. The results of MTs and Transwell experiments showed that hsa_circ_0040809 and hsa_circ_0000467 promoted CRC proliferation and migration. Bioinformatics analysis screened 3 miRNAs (miR-326, miR-330-5p, and miR-330-3p) and 2 mRNAs (*FADS1* and *RUNX1*), and a ceRNA network was constructed. In knockdown of hsa_circ_0040809 HCT-116 cells, the expression of miR-330-3p was significantly upregulated, while *RUNX1* was significantly downregulated. In knockdown of hsa_circ_0000467 HCT-116 cells, the expressions of miR-326 and miR-330-3p were upregulated, while *FADS1*was downregulated.

**Conclusion:** We found that hsa_circ_0040809 and hsa_circ_0000467 were upregulated in CRC tissues and cell lines, and promoted CRC cell progression. A circRNA-miRNA-mRNA network based on hsa_circ_0040809 and hsa_circ_0000467 was constructed.

## Introduction

Colorectal cancer (CRC) is the third most common cancer and the second leading cause of cancer-related death in the world (Sung et al., 2021). Surgical treatment, along with other treatments including chemotherapy, radiotherapy and targeted medical therapy, are primary treatment for CRC. Despite advances in the treatment, the prognosis of CRC patients still is poor. The 5-year survival rate of CRC is approximately 50%, closely related to early diagnosis (Yi et al., 2016; Zhang et al., 2018). Therefore, better understanding of the mechanisms of CRC carcinogenesis and metastasis is critical to identify novel biomarkers for early diagnosis and prognosis of CRC.

Circular RNA (circRNA) is a novel class of non-coding RNA, more stable than linear RNAs due to the lack of 5′ end caps and 3′ poly (A) tails. CircRNAs have been found differentially expressed in various malignant tumors, and therefore been a focus of research as potential biomarkers for cancer. Increasing evidences have shown that circRNAs may function as novel diagnostic and prognostic biomarkers in cancers including CRC (Naeli et al., 2020), gastric cancer (Wang et al., 2019a), ovarian cancer (Shabaninejad et al., 2019), and melanoma (Hallajzadeh et al., 2020). Sponging miRNAs, functioning as the competing endogenous RNA (ceRNA), is one of main mechanisms of circRNAs in cancer. CircSLC8A1 suppressed bladder cancer by sponging miR-130b/miR-494 (Lu et al., 2019a), has_circ_0091570 inhibits the proliferation and invasion of hepatocellular carcinoma by regulating hsa-miR-1307 (Wang et al., 2019b), and circularYAP1 suppresses gastric cancer by sponging miR-367-5p (Liu et al., 2018).

A number of circRNAs have been reported to be differentially expressed in CRC, and play key roles in cancer progression. For example, circDENND4C (Zhang et al., 2020) and circ_0000218 (Pei et al., 2020) exhibit carcinogenic roles in CRC, and hsa_circ_0137008 (Yang et al., 2020) and circ-SMAD7 (Wang et al., 2020) suppress metastasis by sponging miRNAs. More circRNAs need to be found as potential biomarkers for CRC diagnosis and treatment.

In this study, we aimed to seek differentially expressed circRNAs (DEcircRNAs) in CRC, explore their role for CRC and construct a possible competing endogenous RNA (ceRNA) network. We obtained the DEcircRNAs by analyzing the circRNA microarray from Gene Expression Omnibus (GEO). The DEcircRNAs were validated in CRC cell lines and tissues. The roles of DEcircRNAs were detected in CRC cell lines. A potential ceRNA network was constructed by a series of bioinformatics analysis.

## Materials and methods

### CircRNA microarrays and differential expression analysis

CircRNA microarrays of CRC and adjacent normal tissues were collected from GEO datasets (https://www.ncbi.nlm.nih.gov/geo/). We searched circRNA expression profiles for human colorectal carcinoma and adjacent normal tissues using the following search terms: colorectal cancer (All Fields) and [circRNA (All Fields) or circular RNA (All Fields)]. Microarrays with sample counts of less than five were excluded. Datasets from CRC cell lines were excluded. Finally, we selected GSE126094, GSE138589, and GSE142837, which were based on the same platform (GPL19978).

A zero-mean normalization method was performed before differential expression analysis (Bandyopadhyay et al., 2014). R Limma package was used for analysis of differential expression of the circRNAs. |log 2 (fold change [FC])| ≥1 and *p* < 0.05 was used as the criteria for DEcircRNAs. The overlapped DEcircRNAs of the three microarrays were selected by Venn analysis.

### Patients and samples

The inclusion criteria of patients included: adult patients; patients diagnosed as gastric cancer by pathology; without radiation or chemotherapy before operation. Fifteen CRC samples and cancer adjacent tissues were collected from fifteen patients in Fujian Cancer Hospital. These samples were stored in −80°C. The Ethics Committee of Fujian Cancer Hospital approved this study (No. SQ 2022-071-01).

### Cell culture

The human CRC cell line HT29 and HCT 116 were donated from the Fujian Provincial Key Laboratory of Tumor Biotherapy. The FHC normal intestinal mucosal cell line was purchased from American Type Culture Collection (ATCC). All cell lines were validated *via* the STR method. FHC cells were cultured in DMEM cell medium, while HT29 and HCT 116 were cultured in 1,640 cell medium. The methods of cells culture were described previously (Liu et al., 2020).

### Has_circ_0000467 and has_circ_0040809 knockdown

The siRNAs to knockdown has_circ_0000467 and has_circ_0040809 were provided by GenePharma (Shanghai, China). The experimental procedure was conducted according to the manufacturer’s instructions. The knockdown efficiency of has_circ_0000467 and has_circ_0040809 was detected by qRT-PCR.

### RNA extraction and qRT-PCR

CRC and para-carcinoma tissues were ground before RNA extraction. Total RNAs were extracted from cell lines and CRC tiusses by TRIzol reagent (TIANGEN, Beijing, China) according to the manufacturer’s instructions. The purity and concentration of the extracted RNAs were measured using a NanoDrop spectrophotometer (Thermo, Wilmington, DE, United States). The relative expressions of DEcircRNAs were detected by qRT-PCR, described in our previous study (Liu et al., 2020). The relative expressions of the circRNAs were calculated using the 2^−ΔΔCt^ method. The sequences of primers for circRNAs, miRNAs, mRNAs and GAPDH mRNA are shown in Table 1.

**TABLE 1:** Sequences of circRNAs, miRNA, mRNA and GAPDH used for qRT-PCR.

Primer	Sequence (5’to 3’)
hsa_circ_0040809-F	GAAGCCAAATTGCAAGCCCT
hsa_circ_0040809-R	CCACTGCAATCTGAACCACA
hsa_circ_0000467-F	ACCCCACCTACCAAACAATC
hsa_circ_0000467-R	TGGCTTCTTGCTCGTGTACT
hsa_circ_0084615-F	TCAGAGGTGCTTCAAGGAAAAC
hsa_circ_0084615-R	CCCAGCAATGCAATCACCAT
hsa_circ_0000512-F	ATGGCTGAGGTGAGGTGAGT
hsa_circ_0000512-R	GGCCGTGAGTCTGTTCCAAG
BANP-F	TAGTGACAGATGAAGACGAACCT
BANP-R	CTATCCAACCGCAAGCAGATT
SKA3-F	GTGATGCCGAATATACCAACTCTC
SKA3-R	CCAACGAAGTACGATCTTCAACT
hsa-miR-326-F	CCGAATAATCCTCTGGGCCCTTC
hsa-miR-326-R	AGTGCAGGGTCCGAGGTATT
hsa-miR-326-RT	GTCGTATCCAGTGCAGGGTCCGAGGTATTCGCACTGGATACGACCTGGAG
hsa-miR-330-5p-F	CCGAATATTCTCTGGGCCTGTGTC
hsa-miR-330-5p-R	AGTGCAGGGTCCGAGGTATT
hsa-miR-330-5p-RT	GTCGTATCCAGTGCAGGGTCCGAGGTATTCGCACTGGATACGACGCCTAA
hsa-miR-330-3p-F	CTTAGCAAAGCACACGGCCTG
hsa-miR-330-3p-R	AGTGCAGGGTCCGAGGTATT
hsa-miR-330-3p-RT	GTCGTATCCAGTGCAGGGTCCGAGGTATTCGCACTGGATACGACTCTCTG
FADS1-F	TCTGCCTTCAATGACTGGTTC
FADS1-R	GCTTGGACTGGTACTCTATGC
RUNX1-F	CACTGTGATGGCTGGCAATG
RUNX1-R	CTTGCGGTGGGTTTGTGAAG
GAPDH-F	CACCCACTCCTCCACCTTTGA
GAPDH-R	TCTCTCTTCCTCTTGTGCTCTTGC

### Transwell migration experiment

Cell concentration was adjusted to 1×10^6^/ml, and 200 µL of the cell suspension was placed onto the upper chamber of each well on 24-well-Transwell plates. The lower chamber contained medium with 30% fatal bovine serum (FBS). After incubation at 37°C for 24 h, the cells in the upper chamber were wiped off with cotton swabs, and the cells in the other side of chamber membrane were fixed with methanol for 15 min, dried, stained with 0.1% crystal violet, and randomly pictured under inverted microscope (Olympus, Japan). The cells were counted with ImageJ program.

### MTs cell proliferation

Cells were plated into 96-well-plates (4,000 cells/well). After culturing 0–3 days, 20 µl of MTs (Promega, Madison, WI, United States.) were added to each well-followed by incubation at 37°C for 2 h. Cell medium only with serum was used as background control. The absorption of the palates at 490 nm were read on a Bio-Rad (Hercules, CA, United States) plate reader.

### Bioinformatics analysis and construction of a ceRNA network

The miRNA and mRNA data of expressions in CRC were obtained from TCGA database (https://portal.gdc.cancer.gov/). The differentially expressed miRNAs and mRNAs (DEmiRNAs and DEmRNAs, respectively) from TCGA were analyzed by R Limma package. |log 2 [FC]| ≥1.5 and *p* < 0.05 was used to indicate significance.

The circRNA-miRNA interactions were predicted by CircInteractome (https://circinteractome.irp.nia.nih.gov/) (Dudekula et al., 2016). The interactions between mRNAs and miRNAs were predicted by miRDB (Chen and Wang, 2020) and TargetScan (Agarwal et al., 2015). Hub genes were identified by the Cytoscape plugin “cytoHubba.” The evaluation of overall survival according to hub gene expression was performed by Gene Expression Profiling Interactive Analysis (GEPIA). A *p*-value < 0.05 was set as the threshold for significance.

### Statistical analysis

All statistical analyses were performed using IBM SPSS version 23.0 (IBM Corporation, Chicago, IL, United States). Student’s t test was performed for statistical analysis. A *p* < 0.05 was considered statistically significant. Figures were drawn by GraphPad Prism 7 (GraphPad Software, La Jolla, CA, United States).

## Results

### Identification of DEcircRNAs

Three circRNA microarrays (GSE126094, GSE138589, and GSE142837), were selected for further analysis in our study. In the GSE126094 dataset, 1,775 DEcircRNAs were identified, including 11 downregulated and 1,764 upregulated circRNAs. In the GSE138589 dataset, 30 downregulated circRNAs and 156 upregulated circRNAs were identified. A total of 43 DEcircRNAs, including nine downregulated and 34 upregulated circRNAs, were identified in the GSE142837 dataset. Venn diagram analysis was performed to identify overlapping DEcircRNAs among the three datasets. The results revealed four up-regulated circRNAs (hsa_circ_0084615, hsa_circ_0040809, hsa_circ_0000467, and hsa_circ_0000512) in all three datasets. The details of the four up-regulated circRNAs in CRC are presented in Table 2.

**TABLE 2:** Overlapped differentially expressed circRNAs from circRNA microarrays in this study.

circRNA ID	circBase ID	Host gene	GSE126094		GSE138589		GSE142837	
			*P*.Value	Log_2_FC	*P*.Value	Log_2_FC	*P*.Value	Log_2_FC
hsa_circRNA_104634	hsa_circ_0084615	ASPH	0.000	1.544	0.011	1.925	0.042	1.390
hsa_circRNA_101900	hsa_circ_0040809	BANP	0.000	1.621	0.025	1.037	0.000	1.028
hsa_circRNA_101231	hsa_circ_0000467	SKA3	0.000	2.562	0.001	1.787	0.033	1.616
hsa_circRNA_000166	hsa_circ_0000512	RPPH1	0.000	4.524	0.036	1.365	0.021	1.566

### Hsa_circ_0040809 and hsa_circ_0000467 are upregulated in CRC cell lines and tissues

**FIGURE 1:**
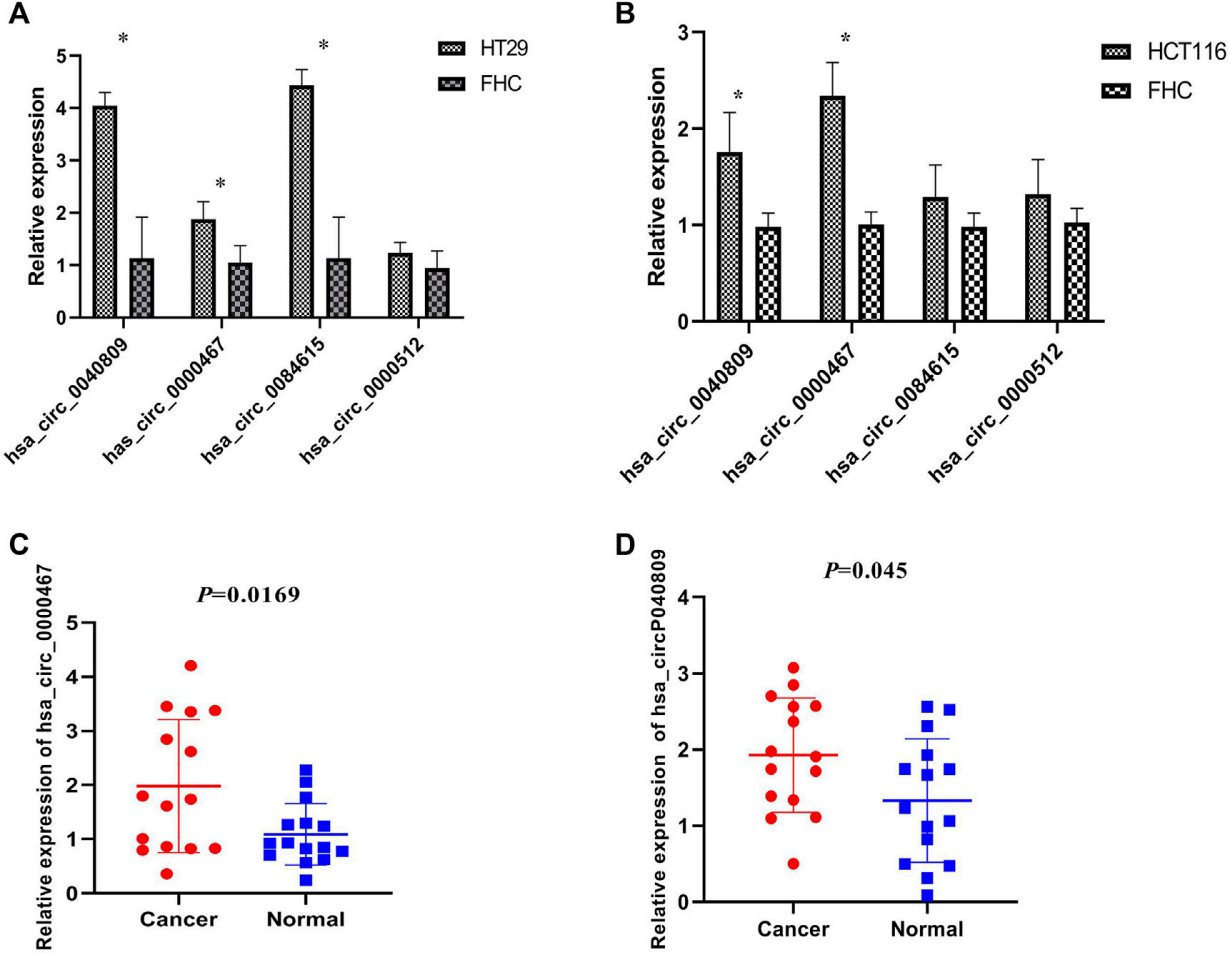
Hsa_circ_0040809 and hsa_circ_0000467 are upregulated in CRC cell lines and tissues. **(A)** The relative expressions of hsa_circ_0040809, hsa_circ_0000467, hsa_circ_0084615, and hsa_circ_0000512 in HT29 and FHC. **(B)** The relative expressions of hsa_circ_0040809, hsa_circ_0000467, hsa_circ_0084615, and hsa_circ_0000512 in HCT116 and FHC. **(C)** The relative expressions of hsa_circ_0000467 in CRC and paracancerous tissues. **(D)** The relative expressions of hsa_circ_0040809 in CRC and paracancerous tissues.

We detected the expression of the above four DEcircRNAs in the HT29, HCT116, and FHC cell lines by qRT-PCR. The results revealed that the expressions of hsa_circ_0084615, hsa_circ_0040809, and hsa_circ_0000467 were up-regulated in HT29 cells (Figure 1A). In HCT116 cell, hsa_circ_0040809 and hsa_circ_0000467 were up-regulated (Figure 1B). Therefore, hsa_circ_0040809 and hsa_circ_0000467 were selected for further study. Their expressions were detected in fifteen CRC samples and cancer adjacent tissues by qRT-PCR. Both hsa_circ_0040809 and hsa_circ_0000467 were upregulated in CRC samples rather cancer adjacent tissues. The results were shown in Figures 1C,D.

### Hsa_circ_0040809 and hsa_circ_0000467 promote CRC proliferation and migration

**FIGURE 2:**
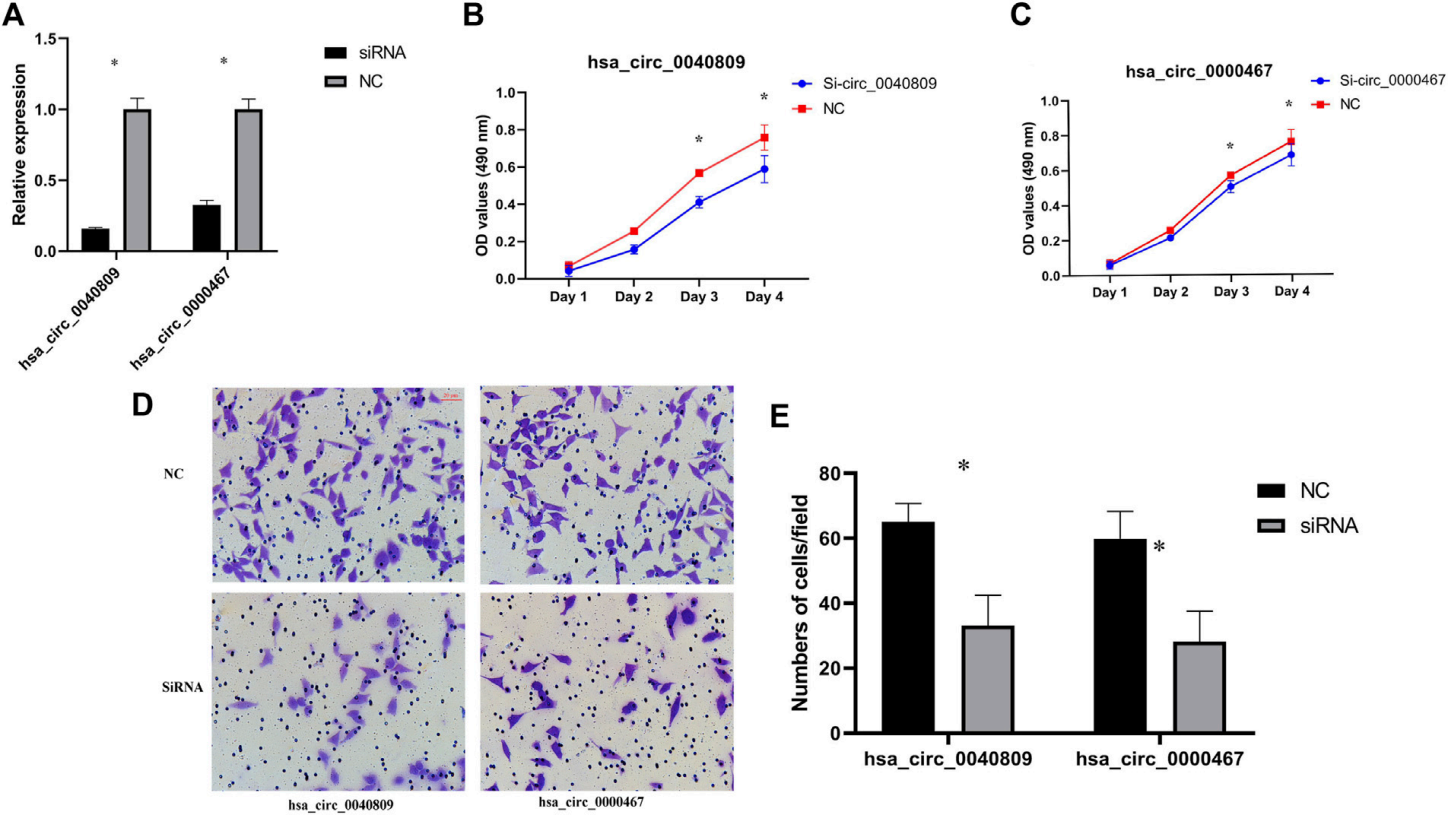
Hsa_circ_0040809 and hsa_circ_0000467 promote HCT116 cells proliferation and migration. **(A)** The efficiency of knockdown hsa_circ_0040809 and hsa_circ_0000467. **(B)** The effect of siRNA-hsa_circ_0040809 on HCT116 cells proliferation. **(C)** The effect of siRNA-hsa_circ_0000467 on the HCT116 cells proliferation. **(D)** Results of siRNA-hsa_circ_0040809 and siRNA-hsa_circ_0000467 Transwell migration of HCT116 cells. **(E)** Numbers of migrated cells/field.

Hsa_circ_0040809 and hsa_circ_0000467 are upregulated in CRC cells and tissues, suggested that hsa_circ_0040809 and hsa_circ_0000467 may promote CRC progression. To test this hypothesis, we designed siRNAs to silence hsa_circ_0040809 and hsa_circ_0000467, which were transfected into HCT116 and HT29. After siRNA transfection to HCT116 cell line, the expressions of hsa_circ_0040809 and hsa_circ_0000467 declined about 70% and 60%, respectively (Figure 2A). Downregulation of hsa_circ_0040809 and hsa_circ_0000467 inhibited HCT116 cells proliferation (Figures 2B,C) and migration (Figures 2D,E).

**FIGURE 3:**
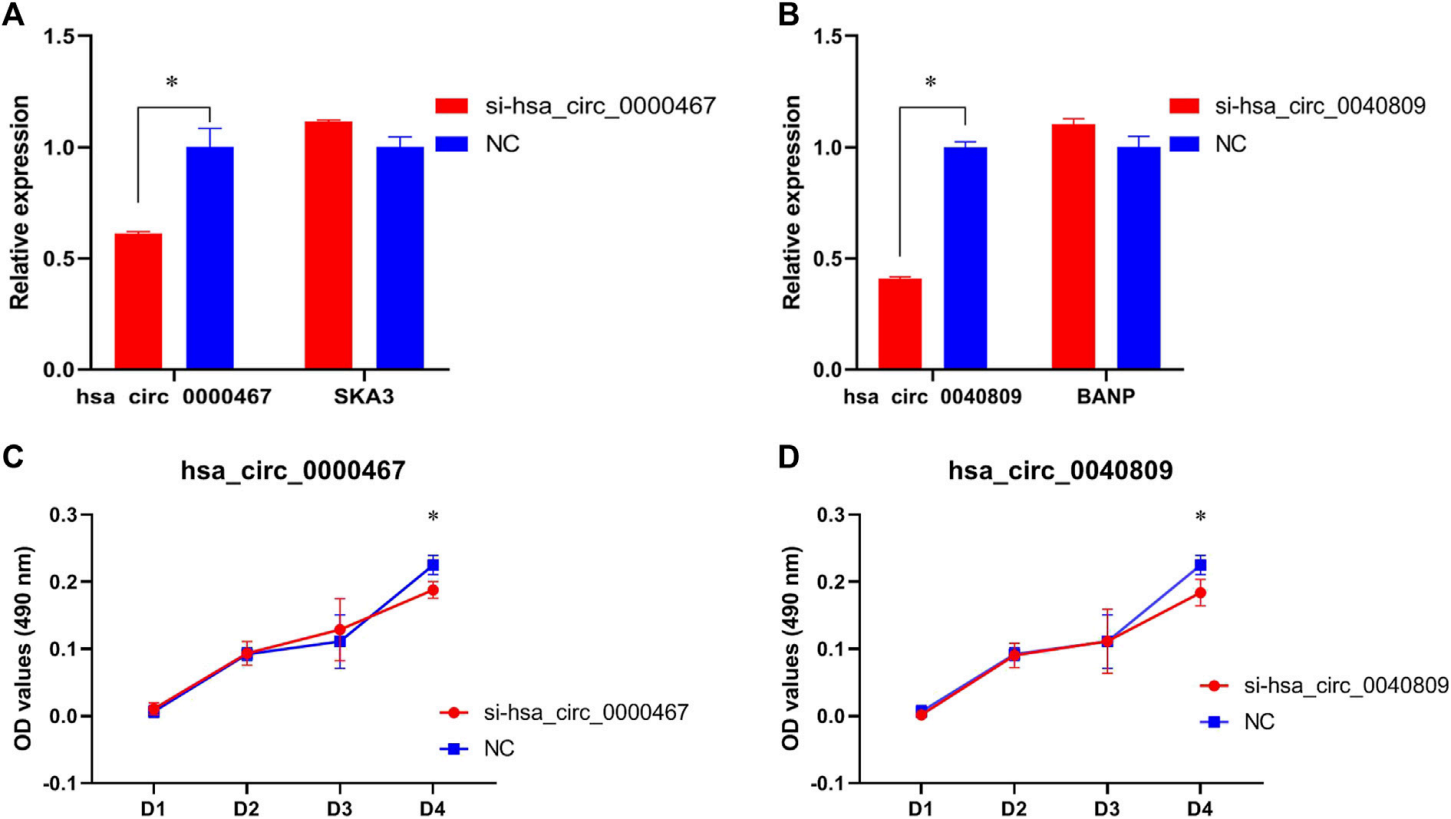
Hsa_circ_0040809 and hsa_circ_0000467 promote HCT29 cells proliferation. **(A)** The relative expressions of hsa_circ_0000467 and host gene SKA3 in siRNA-hsa_circ_0000467 and NC HCT29 cells.**(B)** The relative expressions of hsa_circ_0040809 and host gene BANP in siRNA-hsa_circ_0040809, and NC HCT29 cells. **(C)** The effect of siRNA-hsa_circ_0000467 on HT29 cells proliferation. **(D)** The effect of siRNA-hsa_circ_0040809 on the HT29 cells proliferation.

In HT29 cells, the expressions of hsa_circ_0040809 and hsa_circ_0000467 decreased about 60% and 40%, respectively. Meanwhile, we detected the host genes of hsa_circ_0040809 and hsa_circ_0000467 (BANP and SKA3), and there was no difference between siRNA and NC groups (Figures 3A,B). We detected the roles of hsa_circ_0040809 and hsa_circ_0000467 on HT29 cells proliferation by MTs. And the results showed that downregulation of hsa_circ_0040809 and hsa_circ_0000467 inhibited HT29 cells proliferation (Figures 3C,D).

### Bioinformatics analysis and construction of circRNA-miRNA-mRNA network

To investigate a possible molecular mechanism of promoting CRC progression, we firstly gained the information of hsa_circ_0040809 and hsa_circ_0000467 in CSCD database (http://gb.whu.edu.cn/CSCD/). And we found that hsa_circ_0040809 and hsa_circ_0000467 were exon-circRNAs. That suggested that hsa_circ_0040809 and hsa_circ_0000467 may play a role as a sponge of miRNAs.

**FIGURE 4:**
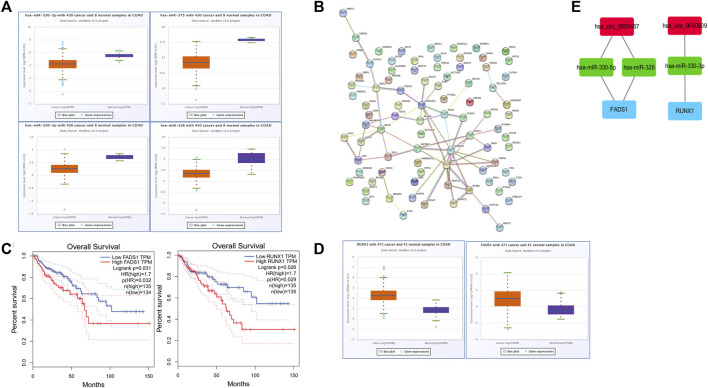
Bioinformatics analysis and aonstruction of circRNA-miRNA-mRNA network. **(A)** The expression of miR-330-3p, miR-330-5p, miR-326, and miR-375 in CRC from ENCORI. **(B)** A protein-protein interaction (PPI) network of 86 mRNAs. **(C)** The expression of *FADS1* and *RUNX1* in CRC from ENCORI. **(D)** Overall survival curves of *FADS1* and *RUNX1* from GEPIA. **(E)** A circRNAs-miRNAs-mRNAs network.

Potential targeted miRNAs of hsa_circ_0040809 and hsa_circ_0000467 were predicted *via* the CircInteractome database. A total of 46 miRNAs were predicted as potential targeted miRNAs. Besides, we identified differentially expressed miRNAs by TCGA database. And 4 of 46 miRNAs (miR-326, miR-330-5p, miR-330-3p, and miR-375) were downregulated. The expression of the miRNAs in CRC is shown in Figure 4A (from ENCORI).

Potential targeted mRNAs of the miRNAs were predicted by TargetScan and miRDB. A total of 780 potential targeted mRNAs were gained. And 86 of 780 mRNA were upregulated in the TCGA database.

The protein-protein interaction (PPI) network of the 86 mRNAs was revealed *via* STRING database. There were 86 nodes and 40 edges in this PPI network (Figure 4B). Hub genes among the 86 mRNAs were identified by the CytoHubba “plugin.” A total of 41 hub genes were identified for further analysis.

The overall survival in CRC according to the expressions of the 41 hub genes was evaluated by GEPIA database. We found that two (*FADS1* and *RUNX1*) of the 41 hub genes were negatively associated with overall survival (Figure 4C). The expressions of *FADS1* and *RUNX1* in CRC is shown in Figure 4D (from ENCORI).

We constructed a possible ceRNA visualized network based on the hub genes with survival prognostic potential, including 2 circRNAs (hsa_circ_0040809 and hsa_circ_0000467), 3 miRNAs (miR-326, miR-330-5p, and miR-330-3p) and 2 mRNAs (*FADS1* and *RUNX1*) (Figure 4E).

### The expression of miRNAs and mRNAs in knockdown of hsa_circ_0040809 and hsa_circ_0000467 CRC cells

**FIGURE 5:**
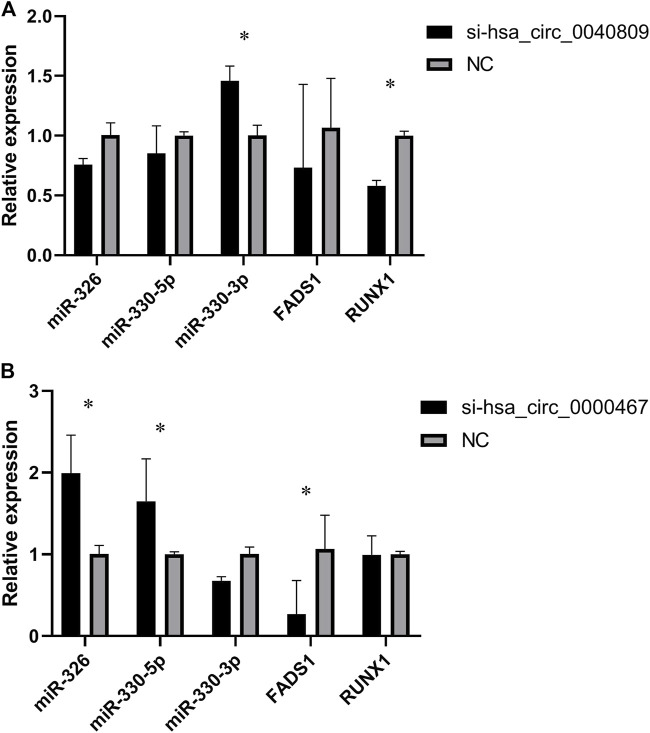
The expression of miRNAs and mRNA in knockdown of hsa_circ_0040809 and hsa_circ_0000467 CRC cells. **(A)** The relative expressions of miR-330-3p, miR-330-5p, miR-375, *FADS1* and *RUNX1* in knockdown of hsa_circ_0040809 CRC cells. **(B)** The relative expressions of miR-330-3p, miR-330-5p, miR-375, *FADS1*, and *RUNX1* in knockdown of hsa_circ_0000467 CRC cells.

To further verify the potential ceRNA networks of hsa_circ_0040809 and hsa_circ_0000467, we detected the expressions of miR-326, miR-330-5p, miR-330-3p, *FADS1*, and *RUNX1* in siRNA-hsa_circ_0040809 or siRNA-hsa_circ_0000467 HCT-116 cells. In siRNA-hsa_circ_0040809 HCT-116 cells, the expression of miR-330-3p was significantly upregulated, while *RUNX1* was significantly downregulated (Figure 5A). In siRNA-hsa_circ_0000467HCT-116 cells, the expressions of miR-326 and miR-330-3p were upregulated, while *FADS1*was downregulated (Figure 5B).

### The expression of miRNAs and mRNAs in CRC tissues

**FIGURE 6:**
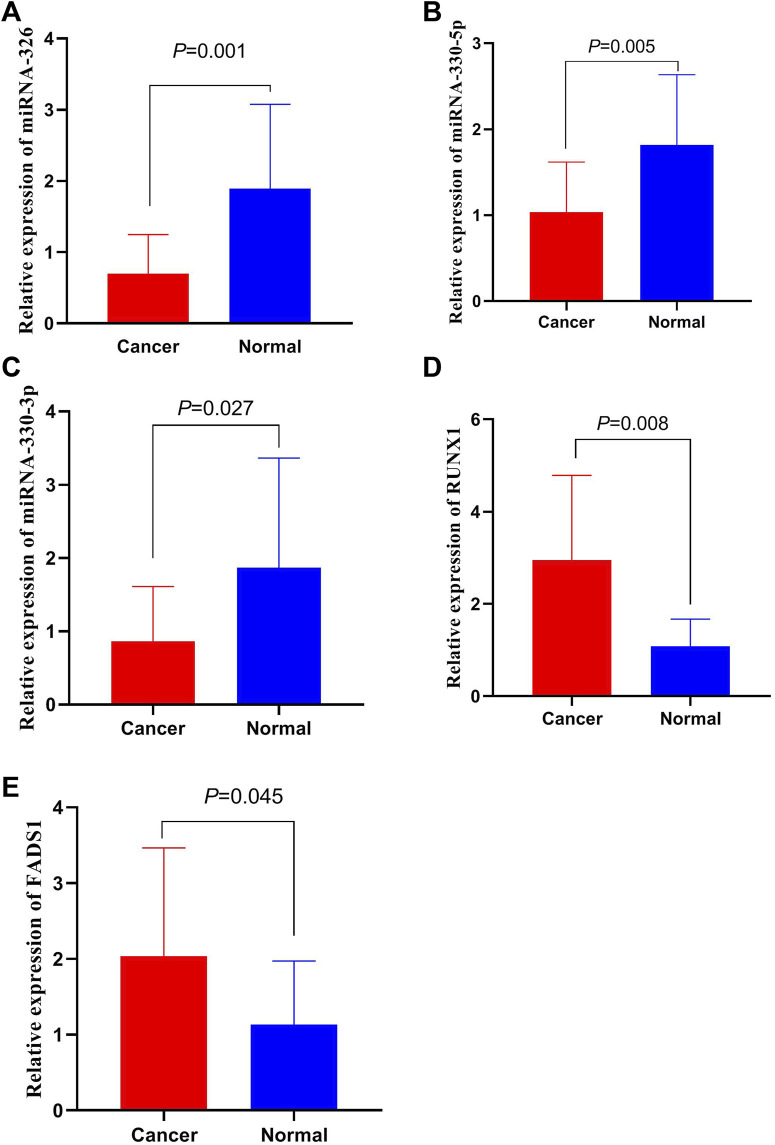
The expression of miRNAs and mRNAs in CRC tissues. **(A)** The expressions of miR-326 in CRC tissues. **(B)** The expressions of miR-330-5p in CRC tissues. **(C)** The expressions of miR-330-3p in CRC tissues. **(D)** The expressions of *RUNX1* in CRC tissues. **(E)** The expressions of *FADS1* in CRC tissues.

We detected the expressions of miR-326, miR-330-5p, miR-330-3p, *FADS1* and *RUNX1* in 15 pairs of CRC and adjacent tissues. The results are revealed in Figure 6. The expressions of miR-326, miR-330-5p, miR-330-3p in CRC tissues are lower than those in adjacent tissues. And *FADS1* and *RUNX1* are highly expressed in CRC tissues rather adjacent tissues. These results are accord with the data from above database.

## Discussion

CircRNAs play a key role in various diseases, including cardiac fibrosis (Yousefi et al., 2020), sepsis (Hashemian et al., 2020), diabetes (Abbaszadeh-Goudarzi et al., 2020), and multiple cancers. In CRC, circCCDC66 promotes colon cancer growth and metastasis (Hsiao et al., 2017), while circPPP1R12A facilitates colon cancer pathogenesis and metastasis (Zheng et al., 2019). Previous studies have reported circRNA-miRNA-mRNA networks in CRC (Song and Fu, 2019; Yuan et al., 2019; Ding et al., 2020). More circRNAs need to be found as potential biomarkers for CRC diagnosis and treatment.

In the present study, we found that hsa_circ_0040809 and hsa_circ_0000467 were upregulated in CRC tissues and cell lines. Further cell experiments show that hsa_circ_0040809 and hsa_circ_0000467 promote CRC cells proliferation and migration. Previous studies have reported that hsa_circ_0040809 facilitates CRC cell proliferation and migration (Mao et al., 2021). Hsa_circ_0000467 is demonstrated to promote the development of gastric cancer (Lu et al., 2019b; Mo et al., 2020).

Acting as ceRNA is a mechanism for regulation of gene expression and a main function of exonic circRNAs in diseases (Tay et al., 2014). CircRNAs share miRNA response element with coding genes (Jafari Najaf Abadi et al., 2019). In CRC, most studies on circRNAs have focused on their functions as ceRNAs. For example, circHIPK3 promotes CRC growth and metastasis by sponging miR-7 (Zeng et al., 2018). Circ_0009361 suppresses CRC progression by sponging miR-582 and targeting APC2 (Geng et al., 2019). In our study, hsa_circ_0040809 and hsa_circ_0000467 were shown as exonic circRNAs by CSCD database. Therefore, we mainly investigated the function as sponging miRNAs. After a series of bioinformatic analysis and screening, we constructed a ceRNA network including two circRNAs (hsa_circ_0040809 and hsa_circ_0000467), three miRNAs (miR-326, miR-330-5p, and miR-330-3p) and two mRNAs (*FADS1* and *RUNX1*).

According to ENCORI database, the expressions of three miRNAs (miR-326, miR-330-5p, and miR-330-3p) were lower in CRC compared with adjacent tissues. They have been reported to suppress cancer development in previous studies. Hsa-miR-326 suppressed cancer development in non-small cell lung cancer (Sun et al., 2016) and prostatic carcinoma (Liang et al., 2018). MiR-330-5p suppressed pancreatic cancer cell (Trehoux et al., 2015) and glioblastoma cell proliferation (Feng et al., 2017). MiR-330-3p was an inhibitor in non-small cell lung cancer (Liu et al., 2015) and osteosarcoma (Zheng et al., 2018).

*FADS1* and *RUNX1* were linked to poor prognosis in CRC by GEPIA database. These genes were associated with the progression of cancers, including CRC. GO enrichment analysis showed that *FADS1* and *RUNX1* were associated with nucleus, positive regulation of transcription from the RNA polymerase II promoter, DNA-templated, peripheral nervous system neuron development, and the nucleoplasm. *RUNX1* was reported to be associated with proliferation, inhibition of apoptosis, and tumor metastasis and invasion (Zhou et al., 2017; Wang et al., 2019c; Xu et al., 2020).

To determine the association of between expressions of miRNAs, mRNAs and circRNAs in the ceRNA network, we detected the expressions of miR-326, miR-330-5p, miR-330-3p, *FADS1*, and *RUNX1* in siRNA-hsa_circ_0040809 or siRNA-hsa_circ_0000467 HCT-116 cells. And the results were accord with the expressions of cicRNAs, miRNAs and mRNAs in ceRNA network. There is no doubt that the results cannot confirm ceRNA mechanism in this study. More efforts will be required to elucidate the role of the identified axes in CRC through *in vitro* and *in vivo* experiments. Nevertheless, the results of this study provide a potential mechanism on that hsa_ circ_0040809 and hsa_circ_0000467 promote CRC proliferation and migration.

## Conclusion

Hsa_ circ_0040809 and hsa_circ_0000467 are upregulated in CRC and promote CRC proliferation and migration. A ceRNA network including hsa_circ_0000467/miR-330-5p/*FADS1*, hsa_circ_0000467/miR-326/*FADS1*, and hsa_circ_0040809/miR-330-3p/*RUNX1* axes is constructed. These axes might be associated with tumorigenesis and prognosis in CRC. Potential novel circRNA biomarkers for CRC were identified and the results provide insights into the underlying mechanisms of CRC pathogenesis. We are furtherly exploring the mechanism of these axes in CRC.

## Data Availability

The datasets presented in this study can be found in online repositories. The names of the repository/repositories and accession number(s) can be found in the article/supplementary material.
